# Supplementary material for: Peripheral Amino Acid Appearance Is Lower Following Plant Protein Fibre Products, Compared to Whey Protein and Fibre Ingestion, in Healthy Older Adults despite Optimised Amino Acid Profile
Source: Nutrients. 2022 Dec 21;15(1):35. doi: 10.3390/nu15010035 (PMC9824653; doi:10.3390/nu15010035)
Supplement: Supplementary file 1 [file nutrients-15-00035-s001.zip › nutrients-2052970-supplementary.pdf]

Article

Peripheral Amino Acid Appearance is Lower Following Plant Protein Fibre Products, Compared to Whey Protein and Fibre Ingestion, in Healthy Older Adults Despite Optimised Amino Acid Profile.

Elena de Marco Castro[1], Giacomo Valli[2], Caroline Buffière[3], Christelle Guillet[3], Brian Mullen[1], Jedd Pratt[2], Katy Horner[1], Susanne Naumann-Gola[4], Stephanie Bader-Mittermaier[4], Matteo Paganini[2], Giuseppe De Vito[2], Helen M Roche[1,5] and Dominique Dardevet[3*] on behalf of the APPETITE Consortium.

[1] UCD Conway Institute & UCD Institute of Food and Health, School of Public Health, Physiotherapy and Sports Science, University College Dublin, Dublin, Ireland.

[2] Neuromuscular Physiology Laboratory, Department of Biomedical Science, University of Padua, Padova, Italy.

[3] Institut National de Recherche pour l'Agriculture, l'Alimentation et l'Environnement, Saint Genès Champanelle, France.

[4] Fraunhofer Institute for Process Engineering and Packaging IVV, 85354 Freising, Germany

[5] The Institute for Global Food Security, School of Biological Sciences, Queen's University Belfast, Belfast, United Kingdom.

* Correspondence: dominique.dardevet@inrae.fr.

Supplementary Materials:

Table S1. A) Sources included in each PPF product blend, and B) PPF products' nutritional composition.[1]

A. Protein sources	protein content (g/ 100 g dry matter basis)	PPF1	PPF2	PPF3
Rice protein isolate	91	0%	0%	18%
Pea protein isolate	88	54%	54%	36%
Pumpkin protein concentrate	62	26%	0%	0%
Soy protein isolate	92	0%	0%	26%
Oat protein concentrate	55	0%	17%	0%
Almond protein concentrate	59	0%	9%	0%
Pea fibre	11	20%	20%	20%

B. Nutritional Composition	PPF1	PPF2	PPF3
Energy (kJ / kcal) *	1552 / 369	1563 / 372	1543 / 367
Moisture (%)	5.3	5.5	5.1
Protein (% dry basis)	65.1	64.0	73.6
Protein (% fresh basis)	61.6	60.5	69.9
Fat (% fresh basis) *	7.7	8.5	4.5
of which saturates (% fresh basis) *	1.6	1.8	1.2
Carbohydrates (% fresh basis) *	4.9	7.7	5.9
Salt (% fresh basis) *	1.4	1.4	1.0

[1]PPF, plant protein fibre. *Calculated from ingredient specifications.

Table S2. Plasma amino acid 3-hr iAUC, C_{max}, and T_{max} in response to test meals containing WPF or PPF products 1-3.[1]

	WPF	PPF1	PPF2	PPF3
ΣAA				
iAUC, μM	163,227 ± 43,854	134,500 ± 41,462	134,500 ± 41,462	134,500 ± 41,462
C_{max}, μM.min	4,280 ± 949	3,797 ± 732	3,835 ± 855	3,937 ± 821
T_{max}, min	107 ± 16	87 ± 32	110 ± 34	117 ± 51
ΣIAA				
iAUC, μM	67,202 ± 18,144	60,905 ± 18,222	60,905 ± 18,222	60,905 ± 18,222
C_{max}, μM.min	1,756 ± 583	1,343 ± 327[a]	1,461 ± 367	1,454 ± 404[b]
T_{max}, min	103 ± 26	103 ± 43	123 ± 35	123 ± 55
ΣBCAA				
iAUC, μM	29,981 ± 9,369	27,360 ± 10,640	27,360 ± 10,640	27,360 ± 10,640
C_{max}, μM.min	954 ± 337	708 ± 155[a]	776 ± 140	858 ± 202[b]
T_{max}, min	100 ± 21	157 ± 33[a]	147 ± 32	150 ± 34
Leucine				
iAUC, μM	8,95 ± 2,904	8,602 ± 3,503	8,602 ± 3,503	8,602 ± 3,503
C_{max}, μM.min	331 ± 116	231 ± 51[a]	254 ± 47	280 ± 69[b]
T_{max}, min	93 ± 23	147 ± 38	130 ± 42	143 ± 44

[1]Values are mean ± SD (n = 10). Data were analysed using a paired sample t-test, except for T_{max}, which was analysed using a Wilcoxon signed-rank test. [a]Indicates a significant difference between from WPF and [b]from PPF1. AA, amino acids; IAA, indispensable AA; BCAA, branched-chain AA; AUC, area under the curve; iAUC, incremental AUC; C_{max}, maximum concentration; T_{max}, time of maximum concentration; WPF, whey protein fibre.
